# Arsenic affects inflammatory cytokine expression in *Gallus gallus* brain tissues

**DOI:** 10.1186/s12917-017-1066-8

**Published:** 2017-06-05

**Authors:** Xiao Sun, Ying He, Ying Guo, Siwen Li, Hongjing Zhao, Yu Wang, Jingyu Zhang, Mingwei Xing

**Affiliations:** College of Wildlife Resources, Northeast Forestry University, Harbin, Heilongjiang Province 150040 China

**Keywords:** Arsenic, NF-κB, Inflammatory cytokines, Brain tissues, Chickens

## Abstract

**Background:**

The heavy metal arsenic is widely distributed in nature and posses a serious threat to organism’s health. However, little is known about the arsenic-induced inflammatory response in the brain tissues of birds and the relationship and mechanism of the inflammatory response. The purpose of this study was to explore the effects of dietary arsenic on the expression of inflammatory cytokines in the brains of *Gallus gallus*.

**Results:**

Seventy-two 1-day-old male Hy-line chickens were divided into a control group, a low arsenic trioxide (As_2_O_3_)-treated (7.5 mg/kg) group, a middle As_2_O_3_-treated (15 mg/kg) group, and a high As_2_O_3_-treated (30 mg/kg) group. Arsenic exposure caused obvious ultrastructural changes. The mRNA levels of the transcription factor nuclear factor-κB (NF-κB) and of pro-inflammatory cytokines, including inducible NO synthase (iNOS), cyclooxygenase-2 (COX-2), and prostaglandin E synthase (PTGEs), in chicken brain tissues (cerebrum, cerebellum, thalamus, brainstem and myelencephalon) on days 30, 60 and 90, respectively, were measured by real-time PCR. The protein expression of iNOS was detected by western blot. The results showed that after being treated with As_2_O_3,_ the levels of inflammatory-related factor NF-κB and pro-inflammatory cytokines in chicken brain tissues increased (*P* < 0.05).

**Conclusions:**

Arsenic exposure in the chickens triggered host defence and induced an inflammatory response by regulating the expression of inflammatory-related genes in the cerebrum, cerebellum, thalamus, brainstem and myelencephalon. These data form a foundation for further research on arsenic-induced neurotoxicity in *Gallus gallus*.

**Electronic supplementary material:**

The online version of this article (doi:10.1186/s12917-017-1066-8) contains supplementary material, which is available to authorized users.

## Background

Arsenic is one of the most toxic substances from the natural environment and is classified as a human carcinogen (Group I) [1]. It is widely distributed in natural sources (earth crust, air, soil), anthropogenic sources (insecticides, feed additives, industrial waste), and in drugs and poisons in both organic and inorganic forms [2]. Arsenic reacts with environmental oxygen, chlorine, and sulfur, generating more toxic, soluble inorganic compounds (AsO_4_^3−^ and AsO_3_^3−^), and posses a serious threat to organism health [3]. Dermal exposure to toxic trivalent or pentavalent arsenic compounds can produce skin cancer, melanosis, and dorsum [4]. Inhalation of arsenic-contaminated air can affect the respiratory system and can cause laryngitis, rhinitis, and pulmonary diseases [5, 6]. Arsenic-contaminated water can be absorbed by the digestive system and leads to gastritis, abdominal pain, and anorexia. Moreover, selenium deficiency can cause muscular dystrophy in various species, arsenic can also damage the liver, kidneys, heart, and reproductive and nervous systems [7–12]. Arsenic can traverse the blood brain barrier and accumulate in different regions of the brain, making it a target organ of arsenic toxicity and suggesting a role for it in neurological diseases [13, 14]. Because of their toxic effects, pollution of the environment with arsenic and arsenic compound attracts public attention [15].

Previous studies showed that the development of toxicity or alteration in cytokines level induced by molybdenum, cadmium, selenium and lead, which was assessed by evaluating mRNA expression and western blot [16–18]. Developing brain tissue is vulnerable to toxic arsenic [19], and arsenic causes histopathological changes to developing brain tissue (unpublished data). Several reports have indicated that acute or chronic exposure to inorganic arsenic causes neural injury [20]. However, little is known regarding whether arsenic-induced neural injury will result in an inflammatory response in the brain tissues of birds. One of the hallmarks of the inflammatory response is the production of pro-inflammatory mediators, which are needed to repair injured tissues [21, 22]. Nuclear transcription factor-κB (NF-κB) is attached to regulatory proteins named inhibitors of κB (IκB) and kept inactive in the cytosol [23]. IκBα and IκBβ are the main proteins involved in NF-κB activation and sustained activation, respectively. NF-κB is activated under inflammation stimulation and then moves to the nucleus, recognizes the promoter region and regulates the transcription of the pro-inflammatory genes inducible NO synthase (iNOS) and cyclooxygenase-2 (COX-2) [24–26]. iNOS is a member of the NOS family and is widely distributed in a diverse number of nerve cells, whereas COX-2 is mainly found in specific neurons [27, 28]. Neither is generally expressed in resting nerve cells, and they are only expressed to take part in inflammatory responses under diverse pathological conditions, such as Alzheimer’s disease, ischaemia, and neurodegenerative disorders [29–31]. iNOS and COX-2 have vital roles in the pathophysiology of inflammation because they produce NO and prostaglandins (PGE), respectively [32–34]. Low concentrations of NO are sufficient to maintain physiological functions; however, elevated NO exerts genotoxic harm on the host [32]. Research on the inflammatory mediators NF-κB, iNOS, COX-2, and prostaglandin E synthase (PTGEs) in chickens treated with arsenic trioxide (As_2_O_3_) may contribute to the understanding of the possible inflammatory mechanisms of heavy metal arsenic in the nervous system of birds.

We explored the effects of arsenic on the mRNA and protein levels of the main inflammatory-related mediators in the brain tissues of Hy-line chickens to answer the question of whether arsenic induced an inflammatory response in chicken brain tissues, specifically the cerebrum, cerebellum, thalamus, brainstem and myelencephalon, by affecting the expression of inflammatory cytokines.

## Methods

### Reagents

RNAiso Plus and PrimeScript™RT reagent Kit were purchased from TaKaRa (Dalian, Liaoning, China). FastStart Universal SYBR Green Master was purchased from Roche (Indianapolis, IN, USA). SDS Lysis Buffer, Enhanced BCA Protein Assay Kit and glyceraldehyde-3-phosphate dehydrogenase (GAPDH) antibody were purchased from Beyotime (Shanghai, China). A horseradish peroxidase (HRP)-labelled goat anti-rabbit IgG was purchased from Beijing Biosynthesis Biotechnology Co., LTD (Beijing, China). The iNOS antibody was kindly provided by Professor Xu (Northeast Agricultural University, China).

### Animals and experimental design

Procedures used in the present study were authorized by the Institutional Animal Care and Use Committee of Northeast Forestry University (Harbin, China) (UT-31; 20 June 2014). The Hy-line chicken models were created according to our previous research [35]. In short, 72 1-day-old healthy Hy-line chickens, purchased from Weiwei Co. Ltd., Harbin, China., were randomly divided into four groups (18 cocks per group): a control group, a low (7.5 mg/kg) As_2_O_3_-treated group (L group), a middle (15 mg/kg) As_2_O_3_-treated group (M group), and a high (30 mg/kg) As_2_O_3_-treated group (H group), which arsenic doses of dietary were daily administration by adding As_2_O_3_ into the food to make supplements uniformed according to the chicken median lethal doses (LD_50_) of 0, 1/80, 1/40, 1/20, respectively. The composition of the diet is: Maize, grains 421 g/kg; Wheat, grains 120 g/kg; Full fat soy 180 g/kg; Pea 100 g/kg; Wheat bran 80 g/kg; Limestone 80 g/kg; Dicalcium phosphate 15 g/kg and Sodium chloride 4 g/kg. This diet met the minimum requirements for energy and nutrients for chicken and without influencing results according to Nisianakis et al. [36]. Food and water were provided ad libitum. During the experiments, all chickens were injected with sodium pentobarbital to abate stress. Six brain tissue samples (cerebrum, cerebellum thalamus, brainstem and myelencephalon) were taken on day 30, 60 and 90, excised and then rinsed with ice-cold sterilized deionized water, and promptly frozen in liquid nitrogen until required.

### Ultrastructural observations

For electron microscopy, brain tissue specimens were fixed with 2.5% glutaraldehyde in 0.1 M sodium phosphate buffer (pH 7.2) for 3 h at 4 °C, washed in the same buffer for 1 h at 4 °C and post-fixed with 1% osmium tetroxide in sodium phosphate buffer for 1 h at 4 °C. The tissues were then dehydrated in a graded series of ethanol starting at 50% ethanol for 10 min at a time and then were immersed twice in propylene oxide. The tissue specimens were embedded in araldite. Ultrathin sections were stained with Mg-uranyl acetate and lead citrate for evaluation using a transmission electron microscope.

### Primers

Primers used in the present study to detect inflammatory cytokine expression in chickens treated with As_2_O_3_ are shown in Additional file 1 [26]. GAPDH was considered a housekeeping gene and was used in this study as an internal reference.

### Total RNA isolation and reverse transcription

Total RNA from chicken brain tissues was isolated using RNAiso Plus (Takara, China). The concentration and purity were analysed by measuring the absorbance at 260 and 280 nm on a spectrophotometer (Ultrospec 1100 pro, Amersham Biosciences, China). The first-strand cDNA was synthesized using the PrimeScriptTMRT reagent Kit (TaKaRa, China) according to the manufacturer’s instructions. The single chain cDNA was diluted tenfold with sterile ddH_2_O and stored at −80 °C before use.

### Quantitative real-time PCR

Quantitative real-time PCR was performed on a BIOER LineGene 9600 Real-Time PCR System (Hangzhou, China). Reactions contained 5 μL of the SYBR Green Master Mix (Roche, USA), 1 μL of diluted cDNA, 1 μL of each primer (10 μM) and 2 μL of ddH_2_O water. The reaction conditions were set at 95 °C for 10 min, followed by 40 cycles of 95 °C for 15 s and 60 °C for 1 min. A single peak could be seen in the melting curve. The relative abundance of mRNA was calculated using the 2^-∆∆Ct^ method and normalized to the mean expression of GAPDH [37].

### Western blot analysis

Total protein from chicken brain tissues was extracted using SDS Lysis Buffer (Beyotime, China). The concentrations of the protein extracts were measured and calculated using the Enhanced BCA Protein Assay Kit (Beyotime, China). Equal amounts of protein from each extract were subjected to 12% SDS-PAGE gel electrophoresis. Separated proteins were transferred to nitrocellulose (NC) membranes in Tris-glycine buffer for 1 h at 100 mA. The NC membranes were blocked with 5% skim milk at 37 °C and 50 rpm for 4 h and incubated overnight with the diluted iNOS primary antibody (1:1000, provided by Dr. Xu) and GAPDH antibody (1:1000, Beyotime, China) followed by a 1 h incubation with a horse-radish peroxidase (HRP)-conjugated goat anti-rabbit IgG (1:5000, Bioss, Beijing) at 37 °C and 50 rpm. The signals were detected by X-ray film (Kodak, USA), and the corresponding protein expression levels were calculated according to greyscale values of the iNOS and GADPH bands.

### Statistical analyses

GraphPad Prism 5 statistical software was used to analyse the data. When a significant value (*P* < 0.05) was obtained by one-way ANOVA, further analysis was carried out. All data showed a normal distribution and passed equal variance testing. Differences between means were assessed using Tukey’s honest significant difference test for post hoc multiple comparisons. Data are expressed as the mean ± SD of 6 observations.

## Results

All treatment groups showed no mortality during our experiments when compared with the controls. We observed no significantly differences between the feed intake, water intake and the body weight of As_2_O_3_-treated group and the control group.

### Ultrastructural changes

**Fig. 1:**
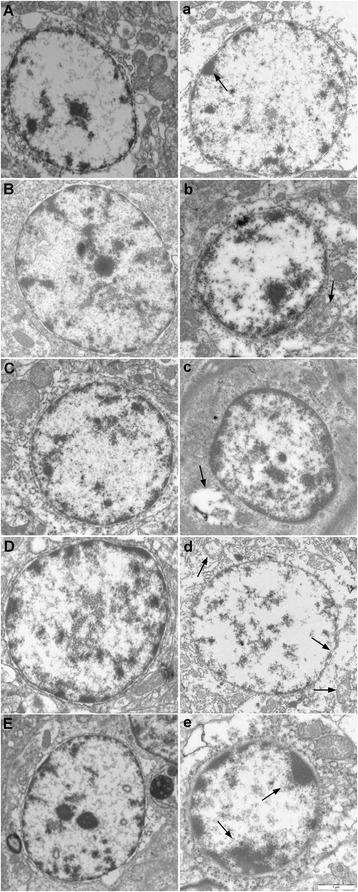
Ultrastructural observations in the brain tissues of chickens. Panels *A*, *B*, *C*, *D* and *E* were the histology of the cerebrum, cerebellum, thalamus, brainstem and myelencephalon tissue in the control group, respectively. Panels **a**, **b**, **c**, **d** and e represented the histology of the cerebrum, cerebellum, thalamus, brainstem, and myelencephalon tissue in the As_2_O_3_ treated groups at 90 d (H groups)

The brain tissues from the control groups showed a normal ultrastructure with cells that had smooth rounded nuclei, intact nuclear membranes, normally distributed chromatin, and integrated mitochondria with normal cristae (Fig. 1A-E). Arsenic treatment caused extensive injury of the brain tissues. The mitochondria in brain tissues of the arsenic groups were swollen and vacuolated with degeneration or loss of cristae. The cells showed typical chromatin condensation and margination, fusion of nuclear membrane, and shrinkage of their nuclei. In addition, the nuclei and organelles of some cells were unclear (Fig. 1a-e).

### Effects of As_2_O_3_ on NF-κB mRNA levels in the brain tissues of chickens

**Fig. 2:**
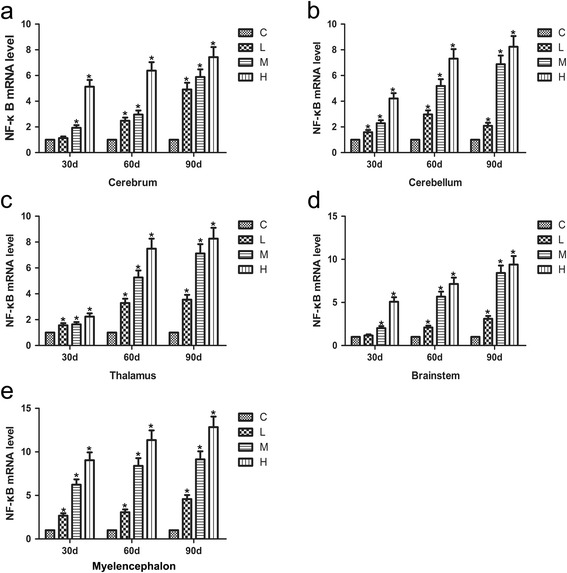
Effects of As_2_O_3_ on the NF-κB mRNA levels in the brain tissues. **a**-**e** represented the NF-κB mRNA levels in the cerebrum, cerebellum, thalamus, brainstem and myelencephalon tissue, respectively. Each value represented the means ± SD of six individuals. The bars with a star at the same sampling time were significantly different (*P* < 0.05)

The NF-κB mRNA levels in the brain tissues treated with As_2_O_3_ are shown in Fig. 2. The NF-κB levels increased in a dose-dependent manner in the cerebrum, cerebellum, thalamus, brainstem and myelencephalon tissues compared with the control group (*P* < 0.05). The NF-κB mRNA levels also increased in a time-dependent manner except the L group of cerebellum, which increased first and then decreased (*P* < 0.05).

### Effects of As_2_O_3_ on the iNOS mRNA levels in the brain tissues of chickens

**Fig. 3:**
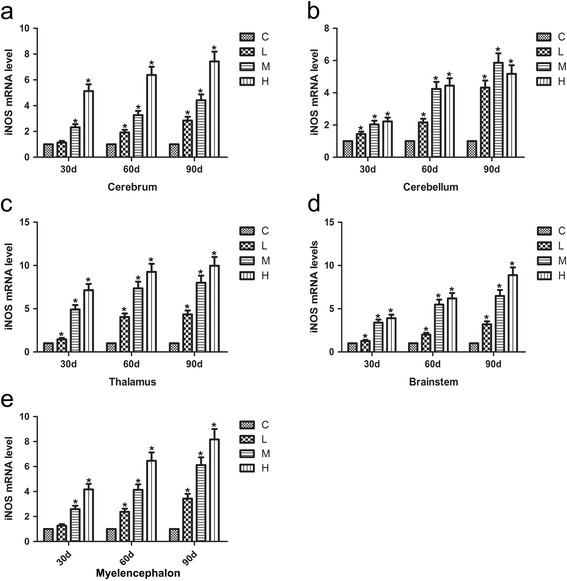
Effects of As_2_O_3_ on the iNOS mRNA levels in the brain tissues. **a-e** represented the iNOS mRNA levels in the cerebrum, cerebellum, thalamus, brainstem and myelencephalon tissue, respectively. Each value represented the means ± SD of six individuals. The bars with a star at the same sampling time were significantly different (*P* < 0.05)

The mRNA levels of iNOS were displayed in Fig. 3. The iNOS mRNA levels were found to be significantly increased in a time-dependent manner after As_2_O_3_ treatment in the cerebrum, cerebellum, thalamus, brainstem and myelencephalon tissues compared with the control chickens (*P* < 0.05). The mRNA levels of iNOS also increased in a dose-dependent manner except for the H group in the cerebellum, which was lower than that of the corresponding M group at 90 d (*P* < 0.05).

### Effects of As_2_O_3_ on the COX-2 mRNA levels in the brain tissues of chickens

**Fig. 4:**
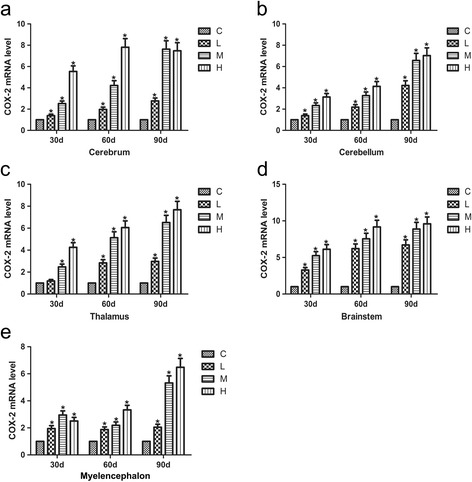
Effects of As_2_O_3_ on the COX-2 mRNA levels in the brain tissues. **a-e** represented the COX-2 mRNA levels in the cerebrum, cerebellum, thalamus, brainstem and myelencephalon tissue, respectively. Each value represented the means ± SD of six individuals. The bars with a star at the same sampling time were significantly different (*P* < 0.05)

The COX-2 mRNA levels in the cerebellum, thalamus and brainstem tissues of chickens treated with As_2_O_3_ increased in a time- and dose-dependent manner compared with the controls (Fig. 4, *P* < 0.05). However, the COX-2 mRNA levels of the H group was lower than that of the M group in the cerebrum at 90 d, whereas the COX-2 mRNA levels in the L and M groups increased in a time- and dose-dependent fashion (*P* < 0.05). The COX-2 mRNA levels of the H group were slightly lower than those of the corresponding M group in the myelencephalon tissue at 30 d, whereas the COX-2 levels were found to be obviously increased in a dose-dependent manner at 60 d and 90 d (*P* < 0.05).

### Effects of As_2_O_3_ on the mRNA levels of PTGEs in chicken brain tissue

**Fig. 5:**
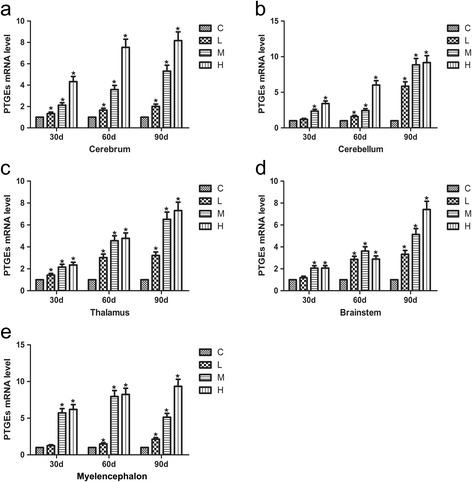
Effects of As_2_O_3_ on the PTGEs mRNA levels in the brain tissues. **a-e** represented the PTGEs mRNA levels in the cerebrum, cerebellum, thalamus, brainstem and myelencephalon tissue, respectively. Each value represented the means ± SD of six individuals. The bars with a star at the same sampling time were significantly different (*P* < 0.05)

The mRNA levels of PTGEs increased in a time- and dose-dependent fashion in the cerebrum, cerebellum, and thalamus of chickens exposed to As_2_O_3_ compared with the controls (Fig. 5, *P* < 0.05). In the brainstem, the L and M groups showed an increasing time- and dose-dependent trend (*P* < 0.05); however, at 60 d, the H group had slightly increased mRNA levels of PTGEs compared with the L group (*P* < 0.05). In the myelencephalon, all groups except the H group showed a dose-dependent increasing trend (*P* < 0.05) in the mRNA levels of PTGEs. The H group had lower mRNA levels of PTGEs at 90 d than at both the 30 and 60 d time points (*P* < 0.05).

### Western blot analysis of the iNOS levels

**Fig. 6:**
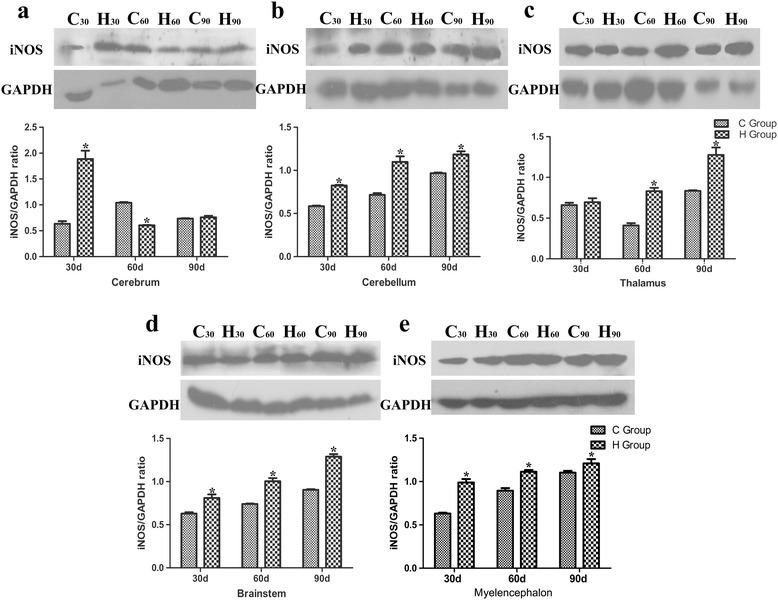
Effects of As_2_O_3_ on the iNOS protein expressions in the brain tissues. **a-e** represented the western blot results of the expression of the iNOS and GADPH proteins and iNOS/GADPH ratio in the cerebrum, cerebellum, thalamus, brainstem and myelencephalon tissue, respectively. C30 and H30 represented the C and H groups at 30 d, respectively, the notations for the C60, H60, C90 and H90 groups are similar. Each value represented the means ± SD of six individuals. The bars with a star at the same sampling time were significantly different (*P* < 0.05)

As_2_O_3_ treatment for a period of 90 days resulted in a significant increase (*P* < 0.05) in the protein expression of iNOS as detected by western blot (Fig. 6). The results revealed that iNOS protein expression was significantly increased in the cerebellum, thalamus, brainstem and myelencephalon tissues in a dose-dependent manner compared with the control group (*P* < 0.05). iNOS protein levels increased at different levels in diverse tissues. Compared with the control group, the iNOS protein levels in the cerebrum tissues of the As_2_O_3_-treated groups increased at 30 d, decreased at 60 d, and slightly increased at 90 d (*P* < 0.05) compared with controls.

## Discussion

As a natural component of the environment, animals easily access to relatively low levels of arsenic by eating food, breathing air, and drinking water [38]. The respiratory, cardiovascular gastrointestinal, haematological, renal, dermal, reproductive, and neurological toxicity of arsenic have been recorded for centuries [2]. Therefore, it is valuable to research how environmental arsenic exposure affects organism health, particularly at low levels. One of the risk factors of neurological toxicity is arsenic. The acute or chronic exposure to inorganic arsenic causes arsenic-associated neurotoxicity in humans that cause behavioural alterations in turn [39, 40]. In this study, we performed an ultrastructure assay of chicken brain tissues and found that arsenic trioxide exposure caused typical features of injury such as fusion of the nuclear membrane, nucleus shrinkage, chromatin condensation, and margination. The As_2_O_3_-induced neural injury could further trigger host defences, such as an inflammatory response. Therefore, we investigated the effect of As_2_O_3_ on the expression of inflammatory cytokines in the brain tissues of chickens.

When organisms are exposed to heavy metals, NF-κB level increases, interact with reactive oxygen species (ROS) [41] and accelerate the generation of inflammatory cytokines [42]. In our study, the expression of NF-κB was assessed by qRT-PCR, and we confirmed that NF-κB expression was significantly increased in a time- and dose-dependent manner except in the cerebrum and brainstem tissues from the As_2_O_3_-treated L group (*P* < 0.05). The expression of NF-κB was similar trend in a time- and dose-dependent manner in five brain tissues. Increased NF-κB activity has been found in the brain tissues of patients with Alzheimer’s disease and takes part in the neurodegenerative process [43]. Our results showed that arsenic activated NF-κB expression in the brain tissues of chickens, which might further induce the expression of other pro-inflammatory genes such as TNF-α, IL-6, iNOS, and COX-2 involved in the inflammatory process.

NOS expression has a wide range of distribution and has been found in endothelial cells, glial cells, neurons, and perivascular nerves [27, 28]. iNOS is a member of the NOS family and is expressed in inflammatory cells and nerve cells including astrocytes and microglia [44–46]. Thus, iNOS is well known to be abundant in brain tissue [47, 48]. In our study, we examined iNOS mRNA and protein levels by qRT-PCR and western blot, respectively. The iNOS mRNA levels were significantly increased in the brain tissues of the As_2_O_3_-treated groups except for the cerebellum tissues of the H group and the cerebrum tissues of the L group (*P* < 0.05), suggesting that iNOS might play a role in inducing brain tissue inflammation upon arsenic exposure. In the cerebrum, there was a fluctuation in protein expression that may have been related to post transcriptional regulation of gene expression [49]. Madrigal et al. reported that the iNOS level could be decreased in the brain cortex of animals treated with an NF-κB inhibitor [50]. This provides further evidence that in our study, the activation of NF-κB through arsenic exposure induced iNOS production in different brain tissues.

In addition, it is believed that COX-2 is of primary importance in the inflammatory response [51]. Several studies have shown that kainic acid can lead to the induction of COX-2 expression and neuronal apoptosis. Excitotoxin induces neuronal death in vitro and is accompanied by a selective elevation in the mRNA level of COX-2. Nonsteroidal anti-inflammatory drugs cause the contents of COX to vary in vivo [52]. These observations demonstrate that the expression of COX-2 may be involved in the pathway leading to neuronal death. COX-2 expression and the subsequent prostaglandin E2 (PTGE2) production are both used as prognostic markers of inflammation [53]. Additionally, these two markers are regarded as targets of therapeutic intervention during the inflammatory response. In the tissues damage, enzymes of iNOS and cyclooxygenase-2 (COX-2) could induce the generation of prostaglandin E synthase (PTGEs) [34]. And following the initiation of COX-2 expression, TNF-α could induce the activation of NF-κB in the COX-2 promoter [54]. Consistent with these previous studies, the mRNA levels of COX-2 and the PTGEs were up-regulated in time- and dose-dependent manners in the brain tissues from As_2_O_3_-treated chickens (*P* < 0.05) compared with the control group, especially myelencephalon. The results illustrated that NF-κB activation also up-regulated the expression of iNOS, COX-2 and the PTGEs to take part in an arsenic-induced inflammatory response in the brain tissues of chickens.

## Conclusion

In conclusion, we demonstrated that arsenic exposure in chickens affected the expression of inflammatory cytokines in their brain tissues. Arsenic could trigger host defence and induce an inflammatory response in the brain tissues of chickens. The mRNA levels of NF-κB, iNOS, COX-2 and PTGEs and the protein levels of iNOS were significantly up-regulated in the brain tissues from As_2_O_3_–treated chickens compared with the controls. The mechanisms of neurotoxicity induced by arsenic could lead to inflammatory response in chicken brain tissues.

## Additional file

Additional file 1: Table S1. Primers used for quantitative real-time PCR. (DOC 33 kb)

## Abbreviations

As_2_O_3_: Arsenic trioxide
COX-2: Cyclooxygenase-2
iNOS: Inducible NO synthase
IκB: inhibitors of κB
NF-κB: Nuclear transcription factor-κB
PTGE2: Prostaglandin E2
PTGEs: Prostaglandin E synthase
ROS: Reactive oxygen species
