# Supplementary material for: Arsenic affects inflammatory cytokine expression in Gallus gallus brain tissues
Source: BMC Vet Res. 2017 Jun 5;13:157. doi: 10.1186/s12917-017-1066-8 (PMC5460324; doi:10.1186/s12917-017-1066-8)
Supplement: Additional file 1: Table S1. — Primers used for quantitative real-time PCR. (DOC 33 kb) [file 12917_2017_1066_MOESM1_ESM.doc]

**Table S1** Primers used for quantitative real-time PCR

| Target gene (chicken) | GenBank accession no. | Primer | Sequence  (5′–3′) | PCR fragment length (bp) |
| --- | --- | --- | --- | --- |
| NF-κB | NM_205134 | Forward | TCAACGCAGGACCTAAAGACAT | 162 |
|  |  | Reverse | GCAGATAGCCAAGTTCAGGATG |  |
| iNOS | NM_204961 | Forward | CCTGGAGGTCCTGGAAGAGT | 82 |
|  |  | Reverse | CCTGGGTTTCAGAAGTGGC |  |
| Cox-2 | NM_001167718 | Forward | TGTCCTTTCACTGCTTTCCAT | 84 |
|  |  | Reverse | TTCCATTGCTGTGTTTGAGGT |  |
| PTGEs | NM_001194983 | Forward | GTTCCTGTCATTCGCCTTCTAC | 115 |
|  |  | Reverse | CGCATCCTCTGGGTTAGCA |  |
| GADPH | K01458 | Forward | AGAACATCATCCCAGCGT | 182 |
|  |  | Reverse | AGCCTTCACTACCCTCTTG |  |

^a^Chicken GADPH gene as a housekeeping gene was used as an internal reference.
